# Editorial: Marine microbial symbioses: host-microbe interaction, holobiont's adaptation to niches and global climate change

**DOI:** 10.3389/fmicb.2024.1416897

**Published:** 2024-05-06

**Authors:** Zhiyong Li

**Affiliations:** ^1^State Key Laboratory of Microbial Metabolism and School of Life Sciences and Biotechnology, Shanghai Jiao Tong University, Shanghai, China; ^2^SJTU Yazhou Bay Institute of Deepsea Sci-Tech, Shanghai Jiao Tong University, Sanya, China

**Keywords:** symbioses, microbiome, interaction, climate change, adaptation

Symbiotic relationships between microbes and marine organisms have been found in a variety of marine ecosystems, ranging from shallow coral reefs to deep-sea hydrothermal vents. Marine microbial symbioses provide a way for holobiont's to survive in a very dynamic environment by changing metabolic pathways, and a possible selective force behind evolution (Li, 2019; Apprill, 2020). This Research Topic brings together 11 articles that highlight the association of microbial symbionts with marine sponges (Esposito et al.; Pérez-Llano et al.; Raijman-Nagar et al.), corals (Nie et al.; Sun et al.; Vimercati et al.; Zhang et al.), polychaete (Menabit et al.), shrimps (Qi et al.) and fishes (Fiedler et al.; Shankregowda et al.), and their adaptation to environment changes, providing novel insights into the marine microbial symbioses in regard to host-microbe interaction, holobiont's adaptation to niches and global climate change. Meanwhile, the crucial need to elucidate the underlying mechanisms governing the interactions between the marine microbial symbionts and their hosts is suggested.

Global warming, ocean acidification and pollutants lead to increasingly serious problems of marine ecosystems (Gong et al., 2019; Li et al., 2023; Wu et al., 2023; Chai et al., 2024). Sponges are one of the major ecosystem engineers on the seafloor. Due to their critical roles in regulating marine element cycles, sponges are an essential model for studying and forecasting the impact of global change on marine organisms (Chai et al., 2024; Liu et al., 2024). The study by Raijman-Nagar et al. compared the response of the sponge *Diacarnus erythraeanus*, a widespread Red Sea sponge, from the shallow and mesophotic reefs, to moderate and acute temperature elevation by measuring physiological parameters and the microbiome composition changes, and found that mesophotic and shallow populations of *D. erythraeanus* were highly tolerant to both moderate and acute heat stress. This study supports the hypothesis that mesophotic coral reefs could serve as thermal refugia for some sponge species.

Corals are important components of coral reef system and easily influenced by ocean warming (Gong et al., 2019; Li et al., 2023; Wu et al., 2023; Xiao et al., 2024). Coral bleaching is often accompanied by structural abnormalities of coral symbiotic microbiota, among which *Vibrio* is highly concerned. Sun et al. verified that *Vibrio fortis* was the primary pathogenic bacterium causing coral bleaching, revealed changes in the microbial community caused by *V. fortis* infection, and provided evidence for the “bacterial bleaching” hypothesis. As a well-known pseudo-persistent environmental pollutant, oxybenzone (BP-3) and its related organic ultraviolet filters have been verified to directly contribute to the increasing mortality rate of corals. Here, the impacts of BP-3 on the symbiotic Symbiodiniaceae *Cladocopium goreaui* were explored by Zhang et al.. Symbiodiniaceae could resist the toxicity of a range of BP-3 through promoting cell division, photosynthesis, and reprogramming amino acid metabolism.

Vimercati et al. characterized algal communities of a mesophotic specialist coral species, *Leptoseris cf. striatus*, along the Saudi Arabian Red Sea coast, and indicated that algal symbionts changed over time at the mesophotic depth. Compared to the knowledge of symbiotic Symbiodiniaceae in coral holobionts (Gong et al., 2019), little is known about bacteria in coral skeletons. *Prosthecochloris*, a marine representative genus of green sulfur bacteria, has been found to be dominant in some coral skeletons. *Prosthecochloris* genomes from the skeleton of the stony coral *Galaxea fascicularis* show specialized metabolic capacities to adapt to the microenvironments of coral skeletons, suggesting the adaptive strategy and population evolution of endolithic *Prosthecochloris* strains in coral skeletons (Nie et al.). A metataxonomic study by Esposito et al. revealed for the first time the presence of Rhizobiaceae bacteria in the sponge *Myxilla rosacea*. Global change scenarios could trigger the expression of fungal virulence genes and unearth new opportunistic pathogens, posing a risk to the health of sponges and severely damaging reef ecosystems. In contrast to sponge prokaryotic symbionts, the importance of fungi in sponge holobionts has been largely overlooked (He et al., 2014). Pérez-Llano et al. postulate that manipulating sponge-associated fungal community may be a new strategy to cope with the threats posed to sponge health by pathogens and pollutants, and anticipate that sponge-derived fungi might be used as novel sponge health promoters and beneficial members of the resident sponge microbiome in order to increase the sponge's resistance.

Host-associated microbiota can influence host phenotypic variation, fitness and potential to adapt to local environmental conditions. Both host evolutionary history and the abiotic/biotic environment can influence the diversity of microbiota. However, our understanding of host-microbiome interactions in natural populations is limited since environmental and host-specific factors remain largely unknown. Here, the results from Shankregowda et al. based on the bacterial diversity analysis of three-spined stickleback (*Gasterosteus aculeatus*) and nine-spined stickleback (*Pungitius pungitius*) suggested that host habitat rather than evolutionary history could explain gut microbiome diversity in sympatric stickleback species. The bacterial colonization of newly hatched fish is important for the larval development and health. To date, little is known about the ontogeny of the early microbiota of fishes. According to Fiedler et al., the skin and gut microbiota of Atlantic salmon are similar, but start diverging during the yolk sac stage. Both the skin and gut microbiota are highly dynamic and underwent major changes throughout the yolk sac stage.

Although many studies have been focused on the microbial populations of benthic and pelagic habitats, little is known about bacteria colonizing tube-dwelling polychaetes. Menabit et al. found that polychaete *Melinna palmata* Grube harbored a distinct bacterial assemblage as compared to their sediments. The deduced functional profiles suggested the prevalence of the amino acid and carbohydrate metabolisms, providing a glimpse on the putative role of bacterial community associated with the *Melinna palmata* Grube host. The genus *Rimicaris* is the dominant organism living in hydrothermal vents, however, little is known about the functions of its intestinal microbes. Qi et al. provided genomic evidence for the first symbiotic *Deferribacterota*, a novel gut symbiont from the deep-sea hydrothermal vent shrimp *Rimicaris kairei*. Cofactors such as biotin, riboflavin, flavin mononucleotide, and flavin adenine dinucleotide synthesized by *R. kairei* gut *Deferribacterota* may assist their host in surviving under extreme conditions, suggesting its long-term coevolution with the host.

## Author contributions

ZL: Writing – original draft, Writing – review & editing.
